# Potentials-Attract or Likes-Attract in Human Mate Choice in China

**DOI:** 10.1371/journal.pone.0059457

**Published:** 2013-04-02

**Authors:** Qiao-Qiao He, Zhen Zhang, Jian-Xin Zhang, Zhi-Guo Wang, Ying Tu, Ting Ji, Yi Tao

**Affiliations:** 1 Key Lab of Animal Ecology and Conservational Biology, Centre for Computational and Evolutionary Biology, Institute of Zoology, Chinese Academy of Sciences, Beijing, China; 2 Group for Psychological Assessment and Psychological Intervention, Key Lab of Mental Health, Institute of Psychology, Chinese Academy of Sciences, Beijing, China; 3 Research Planning, Beijing Baihe Online Technology Co., Ltd., Beijing, China; Umeå University, Sweden

## Abstract

To explain how individuals’ self-perceived long-term mate value influences their mate preference and mate choice, two hypotheses have been presented, which are “potentials-attract” and “likes-attract”, respectively. The potentials-attract means that people choose mates matched with their sex-specific traits indicating reproductive potentials; and the likes-attract means that people choose mates matched with their own conditions. However, the debate about these two hypotheses still remains unsolved. In this paper, we tested these two hypotheses using a human’s actual mate choice data from a Chinese online dating system (called the Baihe website), where 27,183 users of Baihe website are included, in which there are 590 paired couples (1180 individuals) who met each other via the website. Our main results show that not only the relationship between individuals’ own attributes and their self-stated mate preference but also that between individuals’ own attributes and their actual mate choice are more consistent with the likes-attract hypothesis, i.e., people tend to choose mates who are similar to themselves in a variety of attributes.

## Introduction

Two old Chinese adages “lang cai nv mao” and “men dang hu dui” may represent the classic standards of long-term human mate choice in traditional Chinese culture. The adage “lang cai nv mao” means that women should choose talented men as long-term partners, and men should choose young and physically attractive women as long-term partners. The adage “men dang hu dui” means that a couple of partners should have the similar family backgrounds. Clearly, the adage “lang cai nv mao” matches the theoretical framework of evolutionary biology based on potential reproductive success, which is largely founded on Trivers’ [1] theory of parental investment. The theory of parental investment predicts that the relative parental investment should be a key factor for the sexual selection and that the mating strategies of males and females should differ. Many studies have shown that (i) women exhibit a stronger preference than men for traits of ambition, social status, financial wealth and commitment in a partner, i.e. men’s reproductive potential as good providers [2], [3], [4], [5], [6], [7], [8], [9]; and (ii) men exhibit a stronger preference than women for characteristics of youthfulness, health, and physical attractiveness in a partner, i.e. women’s reproductive potential as fertile mates [2], [4], [5], [6], [7], [9], [10], [11]. The adage “men dang hu dui” may imply that similarity gives rise to attraction, as widely found in human society (the assortative mating in a trait-by-trait way). For example, some studies have shown that people would like to choose mates who are similar to themselves in variety of attributes, such as height, weight, religion, race, education, and income, etc. [12], [13]; and that established couples tend to be similar to each other on a lot of dimensions, such as age, race, religion, education, physical attractiveness and personality [14], [15], [16], [17], [18].

In addition to the reproductive potential of an individual’s partner, the stability of the partnership (or duration of the relationship) may also influence the reproductive output of their partnership [19], [20], [21]. According to this perspective, the factors concerning the stability of the partnership should be also important in human mate choice. To establish a stable long-term partnership, individuals should adjust their mate preference according to their own relative quality, other than choose the most preferred partner available [19]. Thus, both sexes should look for the traits they desire in the other sex by offering the desirable traits that they themselves possess [22], [23], [24]. There are some evidences to show that selectivity of mate preference should be conditional on self-perception in Western societies [2], [9], [25]. For example, Waynforth and Dunbar [9] showed that women offering cues of physical attractiveness (and men offering resources) make overall higher demands in lonely hearts advertisements; Bereczkei et al. [2] showed that female offering better physical conditions required higher financial and occupational status in potential mates, and men having more resources made more demands about the potential partner’s physical attractiveness.

Based on undergraduates’ self-reports of mate preferences for various attributes and self-perceptions of their own levels on those attributes, Buston and Emlen [19] investigated two alternative hypotheses regarding the relativistic rule of human mate preference in Western society: (i) individuals relate self-perception on sex-specific indicators of reproductive potential to selectivity of mate preference for sex-specific indicators of reproductive potential in the opposite sex; or (ii) individuals relate self-perception on a certain trait to selectivity of mate preference for the same trait. For these two relativistic rules, the first is called the “potentials-attract” hypothesis by Buston and Emlen [19], which means that individuals prefer partners with reproductive potential similar to their own. The second is called the “likes-attract” hypothesis, which means that individuals prefer partners with traits similar to their own. Obviously, the potentials-attract hypothesis emphasizes the difference between the strategies of the sexes (as the adage “lang cai nv mao” implies), and it is the mechanism implicitly assumed in previous evolutionary studies of conditional human mate choice [2], [9], [25]. On the other hand, the likes-attract hypothesis emphasizes the similarity of the strategies of the sexes [4], [5], as the adage “men dang hu dui” implies. It indicates an assortative mating based on a trait-by-trait way. Buston and Emlen [19] investigated 10 attributes and grouped them into four evolutionarily relevant categories (indicative of wealth and status, family commitment, physical appearance, and sexual fidelity). Their main results showed that in Western society, humans do not use a potentials-attract rule in their choice of long-term partners, but rather a likes-attract rule based on a preference for partners who are similar to themselves across a number of characteristics.

Todd et al. [24] argued that although Buston and Emlen [19] found that the modern human mate choices do not reflect predictions of the potentials-attract hypothesis but instead follow the likes-attract hypothesis, the verbally reported mate preferences do not correspond to actual mate choices [24]. Based on a speed-dating data, Todd et al. [24] also obtained a similar result to Buston and Emlen [19] in a pre-event questionnaire, but they found that the self-reported mate preferences did not predict actual mate choices made during the speed-dating. Todd et al.’s [24] main results showed that in actual mate choices, men chose women based mainly on women’s physical attractiveness but not on their own attributes, whereas women chose men whose overall desirability as a mate matched the women’s self-perceived physical attractiveness. This means that the pattern of actual mate choices can be predicted by potentials-attract hypothesis. Kurzban and Weeden [26] also attained a similar result in analysis of participants’ choices made in speed-dating events.

Buston and Emlen’s [19] study was based on 978 undergraduates’ mate preferences for various attributes and self-perceptions of their own levels on these attributes. In Todd et al.’s [24] study, only 46 participants were invited to take part in a research-oriented speed-dating event, and each couple had only five minutes to talk to each other. We also notice that both of these two studies did not show whether individuals’ “mate choices” are reciprocated and eventually turned into a long-term relationship. However, we think that it should be more important to use data on human’s actual long-term mate choice to test both the potentials- and likes-attract hypotheses in order to understand the rules of modern human mate choice.

In this paper, following Buston and Emlen’s [19] and Todd et al.’s [24] basic idea, we investigated how modern Chinese people choose the long-term partners, or which of the old Chinese adages “lang cai nv mao” and “men dang hu dui” works better as a rule of mate choice in China. Different from Buston and Emlen’s [19] and Todd et al.’s [24] studies, our data was from an online dating system, which is one of the largest online dating websites in China. The website provides a business service for heterosexual people who search for long-term partnerships. Each user needs to create a personal account to share his/her personal information and mate preference. Users can visit other people’s profiles freely, and contact someone easily. Moreover, the successful datings (i.e. dating or married couples) via the website are encouraged to report online (called “the successful dating stories”), so that everyone visiting the website could read these stories. Thus, the data from Baihe website provides us a possibility to test both the potentials-attract and likes-attract hypotheses in human’s actual long-term mate choice, i.e. how individuals’ own traits (or self-perceptions) are translated into their stated mate preference [19] and into actual mate choice, as well as whether individuals’ stated mate preference matches their actual mate choice [24]. Furthermore, this data will also show whether there are some differences between China and Western societies in human mate choice.

### Data and Methods

This study was approved by Animal and Medical Ethics Committee of Institution of Zoology, Chinese Academy of Sciences. All registered users of the Baihe website agreed to the terms of use, in which the website has specified its right to analyze registered user's information and to display the results in media or research publications. Anonymous ID numbers distinguished every user in the data provided by the Baihe website, and neither the names nor any contact information of the users were provided to us so as to protect the privacy of the users. An anonymized data set of this research is freely available upon request from the authors.

The Baihe website had about 27.5 millions (27,432,239) users at the end of 2010, coming from all 34 provinces of China, whereas most of them were from some big cities and developed areas in China, such as Beijing, Shanghai, Guangzhou, Shenzhen, Suzhou and Dongguan, etc. (see the location distribution of users plotted in Figure S1 in Supporting Information (SI)). Users’ average age is 29.1±6.2 (n = 10,984,161) for women and 28.9±6.5 (n = 16,448,078) for men (see the age distribution plotted in Figure S2 in SI). Tables S1–S2 in SI indicates the profile characteristics that users of the website could specify about themselves and their ideal partners. All the information of personal items and mate preference were filled when these users registered and created their personal accounts in Baihe Website.

In this paper, only the users with complete information were considered (for the details, see SI). Our data included only 27,183 users who were from 19 to 60 years old (women: n = 13,677, mean age = 30.40 years, SD = 7.03; men: n = 13,506, mean age = 30.72 years, SD = 6.81), and in these users, there were 590 paired couples who have established the long-term partnerships through the online dating system (women: mean age = 28.90 years, SD = 4.20; men: mean age = 31.59 years, SD = 4.82). The demographic data are shown in Tables S3–S4 in SI.

The attributes regarding users’ personal information and self-perceptions were age, height, income (monthly), education level, self-rated physical attractiveness, desire for children, respectively; and the attributes regarding users’ stated mate preference were age, height, income (monthly) and education level, respectively. The income level (monthly) was rated using 7-point scale. The education level was rated using 4-point scale: 1 for High school or below; 2 for Bachelor; 3 for Master; and 4 for Doctor. The desire for children was rated using 3-point scale: 1 = do not want children; 2 = not sure; and 3 = want children. The users’ physical attractiveness was rated by themselves using a 10-point scale: 1 = extremely unattractive and 10 = extremely attractive.

For the physical attractiveness, some studies (e.g. Ref. [27]) have shown that self-reported (or self-rated) physical attractiveness is not a valid measure of actual physical attractiveness since the correlation between actual physical attractiveness and self-reported physical attractiveness is small (the correlation coefficient is about 0.25). In our data, we also found that mean value of the self-rated physical attractiveness were about 7 (SD ranged from 1.70 to 1.86, see Tables S3–S4 in SI). Although individuals’ self-rated physical attractiveness may not be a good measure of their actual physical attractiveness (since it represents only individuals’ self-estimation for their own physical attractiveness), it has been found that individuals’ self-perception might influence their mate preference as well as actual mate choice [19], [24]. Therefore, we here consider only how individuals’ stated mate preference and their actual mate choice are influenced by their self-rated physical attractiveness.

According to Buston and Emlen [19], the six attributes (i.e. age, height, income (monthly), education level, self-rated physical attractiveness and desire for children) could also be grouped into three evolutionarily relevant categories: physical appearance (height and self-rated physical attractiveness), wealth and social status (income and education), and family commitment (desire for children). However, because of the low internal consistencies between attributes of health and physical attractiveness, Todd et al. [24] analyzed them separately instead of aggregating them into physical appearance domain. We also noticed that internal consistencies of the composites were not reported by Buston and Emlen [19]. Similarly, in our analysis, since correlation coefficients between the two attributes in physical appearance or wealth and social status domain were low (see Table 1, the Pearson’s r ranged from 0.0480 to 0.2977, P<0.0001 [28]–[29]), we analyzed each attribute separately.

**Table 1:** Correlations between attributes in the same evolutionary category for personal items and mate preferences.

Sex		Personal items		Stated preference
		Height and Self-attract^a^	Income and Education	Income and Education
Women	r	0.0808	0.2081	0.2234
	P	**0.0000**	**0.0000**	**0.0000**
Men	r	0.0480	0.2480	0.2977
	P	**0.0000**	**0.0000**	**0.0000**

Women, n = 13677; Men, n = 13506. Significant *P*-values are indicated in bold.

a Self-attract refers to self-rated physical attractiveness.

## Results

As pointed out in the section of introduction, the main goal of this study is to assess which of the potentials-attract hypothesis or likes-attract hypothesis works better in human’s actual long-term mate choice. For both people’s stated mate preference and their actual mate choice, if the potentials-attract hypothesis works, women’s attributes in physical appearance should correlate positively to men’s attributes in wealth and status and in family commitment; and, alternatively, if the likes-attract hypothesis works, both women’s and men’s attributes should be significantly positively correlated to the same attributes in their stated mate preference and to their partners’ same attributes in actual mate choice. However, if both potentials-attract and likes-attract hypotheses are supported, the coefficients of determinants of different regressions were compared (partial R^2^) so as to determining which hypothesis is better supported. Our main results are shown below.

### Individuals’ Own Attributes and their Stated Mate Preference

To assess how individuals’ own attributes (or self-perceptions) are translated into their stated mate preference, we calculated a series of multivariate linear regressions (MLR) in which each of the attributes in individuals’ stated mate preference was regressed on all of their own attributes for women and men separately. Following Buston and Emlen’s [19] data analysis strategy (see also Ref. [24]), we also calculated a series of univariate linear regression for women and men separately, in which each of the attributes in individuals’ stated mate preference was individually regressed on each of their own attributes (see SI).

**Figure 1:**
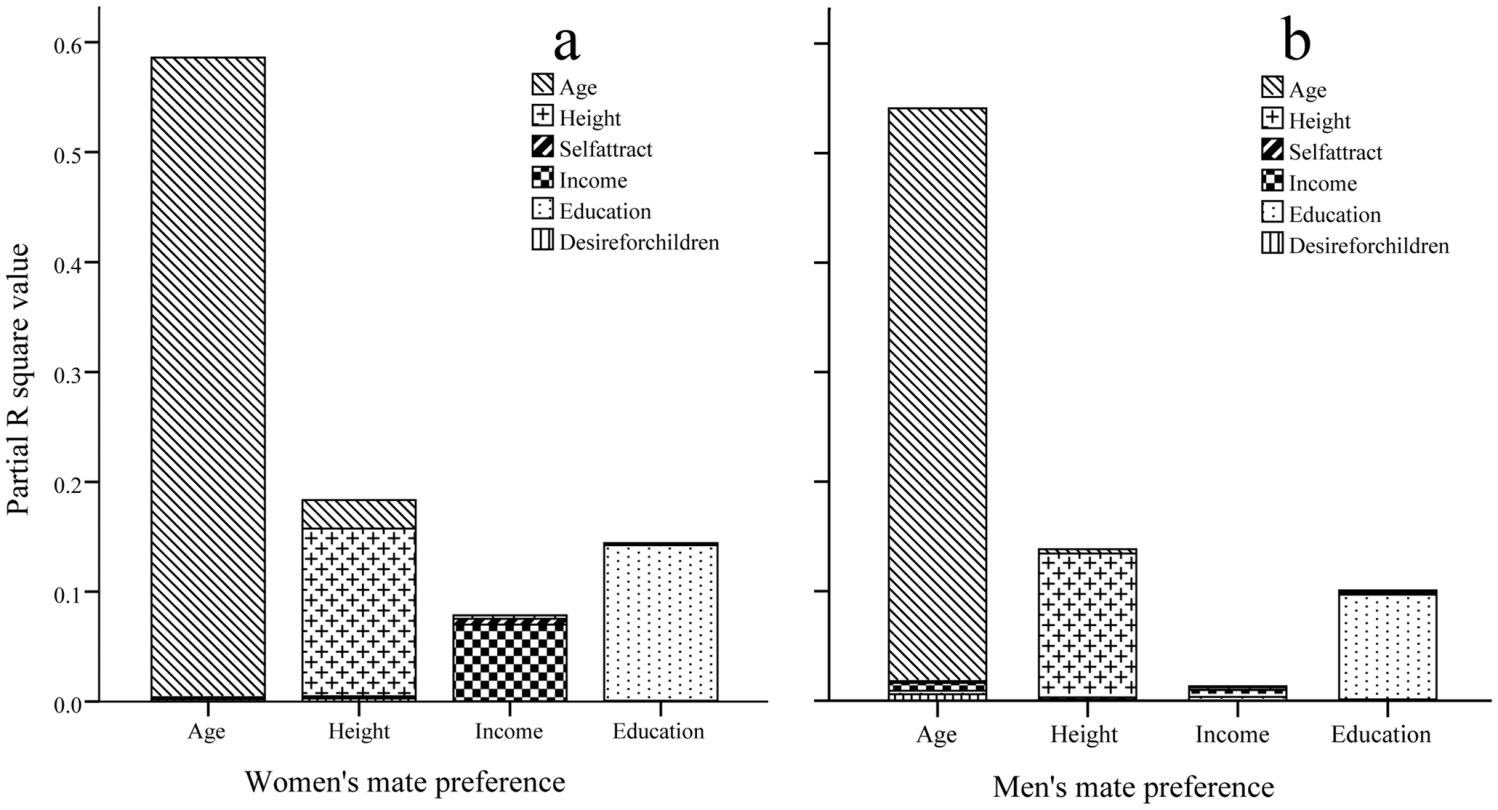
Partial R^2^ values in MLR: Regression of individuals’ stated mate preference on their own attributes. Two panels (a) and (b) indicate women and men.

For the MLR analysis of our data including 27,183 users, i.e. the stated mate preference for each of the four attributes (age, height, income and education) was regressed on users’ own six attributes (age, height, self-rated physical attractiveness, income, education, and desire for children), the results are shown in Table 2 and Figure 1a for women and in Table 3 and Figure 1b for men. For the regressions of women’s stated mate preference on their own attributes (see Table 2 and Figure 1a), there were 19 significant relationships out of 24, showing preliminary support for both hypotheses, in which the highest 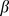-values (or coefficients of determinants, i.e. partial R^2^-values) were consistently those between the same attributes in personal items and mate preference (with P<0.0001), i.e. (i) 58.21% of the variation in women’s stated age preference could be explained by their own age; (ii) 15.25% of the variation in women’s stated height preference could be explained by their own height, whereas 2.6% of the variation in women’s stated height preference could be explained by their own age (with negative 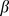-value, P<0.0001); (iii) 6.98% of the variation in women’s stated income preference could be explained by their own income; and (iv) 14.16% of the variation in women’s stated education preference could be explained by their own education. However, it is easy to see that on average 0.41% of the variation in women’s stated income preference could be explained by their own height and their own self-rated physical attractiveness (with P<0.0001) (i.e. the potentials-attract hypothesis could be only partially supported). There are also some statistical results which cannot be predicted by either potentials-attract or likes-attract hypotheses (e.g. the positive correlation between women’s own income and their height preference, and the negative correlation between women’s own education level and their age preference). But these effects are rather small, therefore, only few variations in the correlations between individuals’ own attributes and their stated mate preference can be explained by these effects.

**Table 2:** Results from MLR of women’s stated mate preference regressed on their own attributes.

Women		Age	Height	Income	Education
Age	Beta weight	0.8826	−0.1865	0.0083	0.0010
	P	**0.0000**	**0.0000**	0.3772	0.9147
Height	Beta weight	0.0086	0.3951	0.0560	0.0130
	P	**0.0173**	**0.0000**	**0.0000**	0.1010
Self-attract^a^	Beta weight	−0.0012	0.0163	0.0735	0.0336
	P	0.7490	**0.0375**	**0.0000**	**0.0000**
Income	Beta weight	0.0079	0.0161	0.2763	0.0284
	P	**0.0360**	**0.0446**	**0.0000**	**0.0005**
Education	Beta weight	−0.0407	0.0424	0.0185	0.3889
	P	**0.0000**	**0.0000**	**0.0280**	**0.0000**
Desire for children	Beta weight	−0.0531	−0.0601	0.0125	0.0324
	P	**0.0000**	**0.0000**	0.1751	**0.0003**
**Adjusted R^2^**		0.8240	0.1980	0.0970	0.1650
**P**		**0.0000**	**0.0000**	**0.0000**	**0.0000**

N = 13677. Significant *P*-values are indicated in bold.

a Self-attract refers to self-rated physical attractiveness.

**Table 3:** Results from MLR of men’s stated mate preference regressed on their own attributes.

Men		Age	Height	Income	Education
Age	Beta weight	0.7802	−0.0685	0.0380	−0.0224
	P	**0.0000**	**0.0000**	**0.0000**	**0.0099**
Height	Beta weight	0.0102	0.3673	0.0449	0.0371
	P	0.0634	**0.0000**	**0.0000**	**0.0000**
Self-attract^a^	Beta weight	−0.0393	0.0172	0.0202	0.0192
	P	**0.0000**	**0.0328**	**0.0193**	**0.0189**
Income	Beta weight	−0.0916	0.0464	0.0846	0.0480
	P	**0.0000**	**0.0000**	**0.0000**	**0.0000**
Education	Beta weight	−0.0590	0.0317	0.0621	0.3251
	P	**0.0000**	**0.0001**	**0.0000**	**0.0000**
Desire for children	Beta weight	−0.0790	0.0046	0.0027	0.0362
	P	**0.0000**	0.5724	0.7548	**0.0000**
**Adjusted R2**		0.6010	0.1510	0.0210	0.1240
**P**		**0.0000**	**0.0000**	**0.0000**	**0.0000**

N = 13506. Significant *P*-values are indicated in bold.

a Self-attract refers to self-rated physical attractiveness.

Similarly, for the regressions of men’s stated mate preference on their own attributes (see Table 3 and Figure 1b), there were 21 significant relationships out of 24. Here, we also found that all the same attribute pairs have the highest 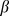-values (i.e. age vs. age, height vs. height, income vs. income and education vs. education, with P<0.0001), i.e. (i) 52.29% of the variation in men’s stated age preference could be explained by their own age, whereas on average only 0.45% of the variation in men’s stated age preference could be explained by their own self-rated physical attractiveness, income, education and desire for children (with negative 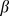-value, P<0.0001); (ii) 13.12% of the variation in men’s stated height preference could be explained by their own height; (iii) 0.62% of the variation in men’s stated income preference could be explained by their own income, whereas on average 0.22% of the variation in men’s stated income preference could be explained by their own age, height and education (with P<0.0001); and (iv) 9.56% of the variation in men’s stated education preference could be explained by their own education, whereas on average only 0.15% of the variation in men’s stated education preference could be explained by their own height, income and desire for children (with P<0.0001).

For both women and men, the similar results were also obtained using the analysis of univariate linear regression for women and men separately (see Table S5 for women and Table S6 for men in SI).

The main results in this subsection should be considered to be more consistent with the likes-attract hypothesis, i.e. similar to the results of Buston and Emlen [19], and also similar to Todd et al.’s [24] finding in their pre-event questionnaires.

### Individuals’ Own Attributes and their Actual Mate Choice

**Figure 2:**
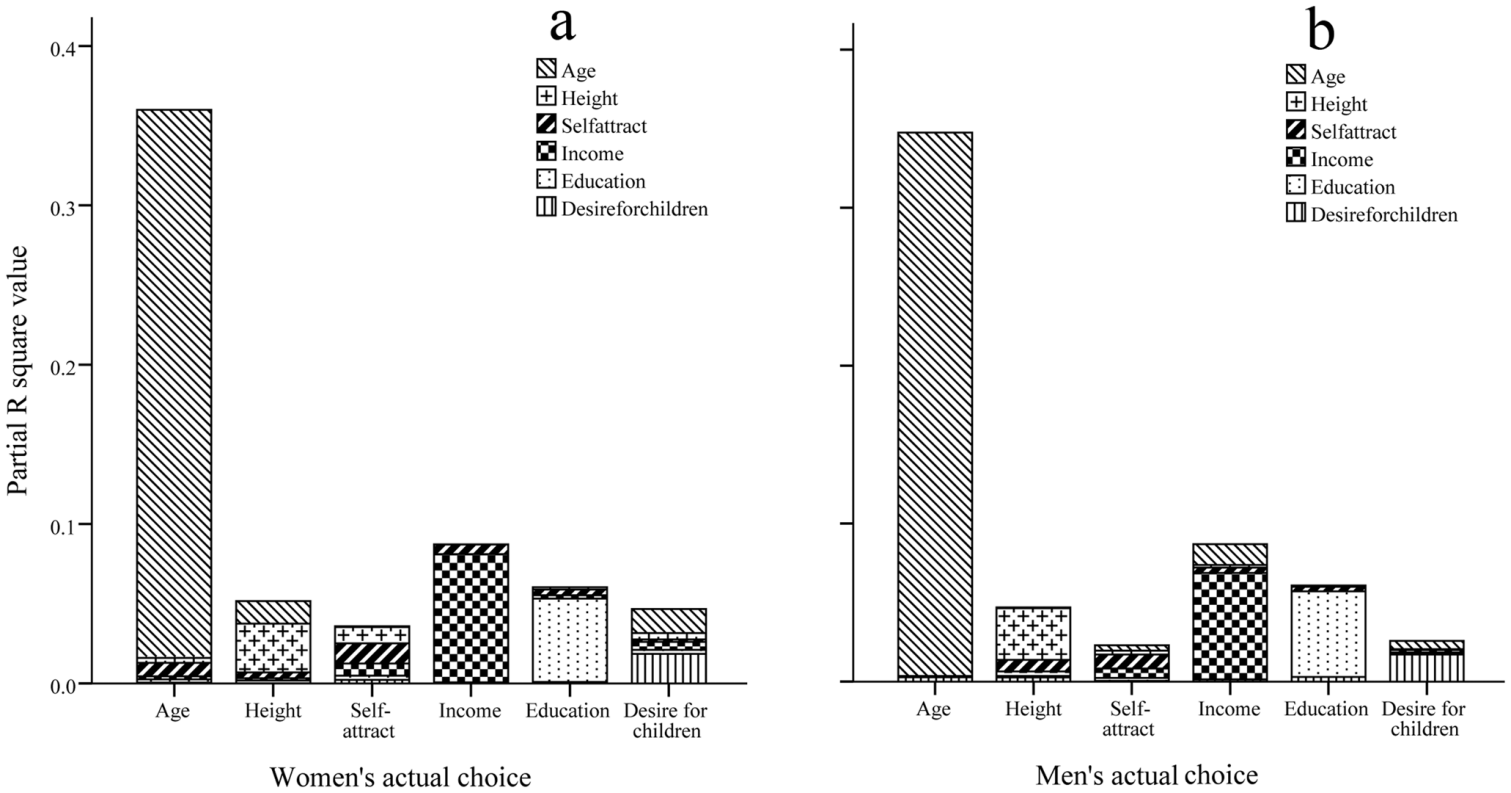
Partial R^2^ values in MLR: Regression of individuals’ actual mate choice on their own attributes. Two panels (a) and (b) indicate women and men.

As pointed out by Todd et al. [24], for both potentials-attract and likes-attract hypotheses, a more challenging question is how individuals’ own attributes affect their actual mate choice. Using the data of 590 couples who have established the long-term partnerships via the website, a series of MLR was calculated, in which each of attributes in users’ actual mate choice was regressed on all of their own attributes. The results are shown in Tables 4 and 5 and Figures 2a and 2b representing partial R^2^ values for women and men, respectively.

**Table 4:** Results from MLR of women’s actual mate choice regressed on their own attributes.

Women		Age	Height	Self-attract^a^	Income	Education	Desire for children
Age	Beta weight	0.6081	−0.1238	0.0234	0.0064	−0.0058	−0.1279
	P	**0.0000**	**0.0032**	0.5789	0.8749	0.8882	**0.0024**
Height	Beta weight	−0.0544	0.1775	−0.1027	0.0093	−0.0387	0.0637
	P	0.0962	**0.0000**	**0.0130**	0.8143	0.3395	0.1209
Self-attract^a^	Beta weight	0.0932	−0.0613	0.1134	0.0791	0.0596	−0.0405
	P	**0.0043**	0.1332	**0.0060**	**0.0465**	0.1406	0.3222
Income	Beta weight	0.0473	0.0396	0.0934	0.3010	0.0492	0.0752
	P	0.1669	0.3548	**0.0308**	**0.0000**	0.2460	0.0802
Education	Beta weight	−0.0467	0.0424	0.0530	−0.0050	0.2378	−0.0522
	P	0.1629	0.3118	0.2102	0.9031	**0.0000**	0.2143
Desire for children	Beta weight	−0.0237	0.0098	−0.0476	0.0273	0.0302	0.1385
	P	0.4692	0.8120	0.2505	0.4937	0.4577	**0.0008**
**Adjusted R^2^**		0.3910	0.0450	0.0280	0.0950	0.0610	0.0390
**P**		**0.0000**	**0.0000**	**0.0010**	**0.0000**	**0.0000**	**0.0000**

N = 590. Significant *P*-values are indicated in bold.

a Self-attract refers to self-rated physical attractiveness.

**Table 5:** Results from MLR of men’s actual mate choice regressed on their own attributes.

Men		Age	Height	Self-attract^a^	Income	Education	Desire for children
Age	Beta weight	0.6166	−0.0220	0.0615	0.1208	0.0031	−0.0763
	P	**0.0000**	0.6047	0.1519	**0.0033**	0.9409	0.0757
Height	Beta weight	−0.0091	0.1852	−0.0499	0.0410	0.0304	−0.0064
	P	0.7850	**0.0000**	0.2318	0.3045	0.4575	0.8776
Self-attract^a^	Beta weight	−0.0116	−0.0870	0.0963	0.0591	0.0534	−0.0251
	P	0.7240	**0.0340**	**0.0200**	0.1347	0.1879	0.5437
Income	Beta weight	−0.0229	0.0563	0.0812	0.2763	0.0030	0.0410
	P	0.5080	0.1911	0.0615	**0.0000**	0.9444	0.3447
Education	Beta weight	−0.0166	−0.0337	0.0471	0.0091	0.2445	0.0359
	P	0.6271	0.4267	0.2719	0.8241	**0.0000**	0.4019
Desire for children	Beta weight	−0.0524	0.0522	−0.0190	0.0362	−0.0560	0.1334
	P	0.1123	0.2041	0.6462	0.3612	0.1691	**0.0014**
**Adjusted R^2^**		0.3810	0.0390	0.0230	0.1070	0.0590	0.0210
**P**		**0.0000**	**0.0000**	**0.0040**	**0.0000**	**0.0000**	**0.0060**

N = 590. Significant *P*-values are indicated in bold.

a Self-attract refers to self-rated physical attractiveness.

For the regressions of women’s actual mate choice on their own attributes (see Table 4 and Figure 2a), there were 12 significant relations (out of 36), in which the highest 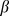-values (or partial R^2^-values) were consistently those between the same attributes, i.e. (i) women’s own age could explain 34.40% of the variation in their partners’ age (with P<0.0001), and it could also explain 1.42% of the variation in their partners’ height (with negative 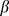-value, P = 0.0032), and 1.52% of the variation in their partners’ desire for children (with negative 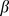-value, P = 0.0024); (ii) women’s own height could explain 3.06% of the variation in their partners’ height (with P<0.0001), and it could also explain 1.02% of the variation in their partners’ self-rated physical attractiveness (with negative 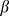-value, P = 0.0130); (iii) women’s own self-rated physical attractiveness could explain 1.26% of the variation in their partners’ self-rated physical attractiveness (with P = 0.0060), and it could also explain 0.85% of the variation in their partners’ age (with P = 0.0043) and 0.61% of the variation in their partners’ income (with P = 0.0465); (iv) women’s own income could explain 8.04% of the variation in their partners’ income (with P<0.0001), and it could also explain 0.77% of the variation in their partners’ self-rated physical attractiveness (with P = 0.0308); (v) women’s own education could explain 5.23% of the variation in their partners’ education (with P<0.0001); and (vi) women’s own desire for children could explain 1.85% of the variation in their partners’ desire for children (with P = 0.0008). Similar to the analysis in the previous subsection, we also noticed that women’s own income could partially explain the variation in their partners’ self-rated physical attractiveness (i.e. women’s own income could explain 0.77% of the variation in their partners’ self-rated physical attractiveness). Clearly, this result was not predicted by either potentials-attract or likes-attract hypotheses. However, its effect was also quite small.

For the regressions of men’s actual mate choice on their own attributes (see Table 5 and Figure 2b), there were only 8 significant relationships out of 36. The pattern was also more consistent with the likes-attract hypothesis, since the highest 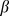-values (or partial R^2^-values) were also those between the same attributes, i.e. (i) men’s own age could explain 34.39% of the variation in their partners’ age (with P<0.0001), and it could also explain 1.32% of the variation in their partners’ income (with P = 0.0033); (ii) men’s own height could explain 3.27% of the variation in their partners’ height (with P<0.0001); (iii) men’s own self-rated physical attractiveness could explain 0.90% of the variation in their partners’ self-rated physical attractiveness (with P = 0.0200), and it could also explain 0.74% of the variation in their partners’ height (with negative 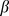-value, P = 0.0340); (iv) men’s own income could explain 6.74% of the variation in their partners’ income (with P<0.0001); (v) men’s own education could explain 5.42% of the variation in their partners’ education (with P<0.0001); and (vi) men’s own desire for children could explain 1.72% of the variation in their partners’ desire for children (with P = 0.0014).

We also calculated a series of univariate linear regression for women and men separately (see Ref. [19], [24]), in which each of users’ own attributes was individually regressed to each of their partners’ attributes. The results were similar to the MLR analysis (see Table S7 in SI).

Basically, the main results in this subsection are also more consistent with the likes-attract hypothesis. This means that for the relationship between individuals’ own attributes and their actual mate choice, people tend to chose mates who were similar to themselves in variety of attributes.

### Individuals’ Stated Mate Preference and their Actual Mate Choice

To assess whether the actual mate choice of both women and men were consistent with their stated mate preference, also using the data of 590 paired couples and following Todd et al. [24], a series zero-order Pearson correlation analysis between individuals’ stated mate preference and their actual mate choice on each of same attribute pairs (i.e. age vs. age, height vs. height, education vs. education, and income vs. income) were calculated for women and men separately. The results are shown in Table 6, in which the correlation coefficients are ranged from 0.122 to 0.440 (with P-values ranged from 0.0029 to smaller than 0.0001). Different from Todd et al.’s [24] result, our analysis showed that for both women and men their actual mate choice matches their stated mate preference.

**Table 6:** Correlations between stated mate preference and actual mate choice for both men and women.

Sex		Actual partners’ characteristics			
		Age	Height	Income	Education
Women	r	0.4154	0.1864	0.1222	0.2684
	P	**0.0000**	**0.0000**	**0.0029**	**0.0000**
Men	r	0.4399	0.2080	0.2373	0.2337
	P	**0.0000**	**0.0000**	**0.0000**	**0.0000**

N = 590 women and 590 men. Significant *P*-values are indicated in bold.

## Discussion

In this study, using the data from a Chinese online dating website, we investigated patterns of human mate choice in China. Our main goal was to show whether the modern Chinese human mate choice can be predicted by the likes-attract hypothesis or potentials-attract hypothesis. Our study is different from the previous studies [19], [24], as it was based on the data of human’s actual mate choice. Following the basic idea in the previous studies [19], [24], we analyzed the relationships between individuals’ own attributes and their stated mate preferences, between individuals’ own attributes and their actual mate choices, and between individuals’ stated mate preferences and their actual choices. Basically, our main results support the likes-attract hypothesis more than the potentials-attract one, i.e. people tend to choose mates who are similar to themselves in a variety of attributes.

Our study provides an example to test the likes-attract and potentials-attract hypotheses in Eastern society. Our main results imply that the likes-attract rule should work better than the potentials-attract rule in human mate preference for long-term mating in both Western and Eastern societies. However, our results about the actual mate choice contradict Todd et al.’s [24] results based on the speed dating. Our analysis was more consistent with the likes-attract hypothesis in human’s actual mate choice. Using the data of the paired couples, we show not only how individuals’ own attributes are translated into their stated mate preference, but also individuals’ actual mate choices could be predicted by the likes-attract hypothesis more than the potentials-attract hypothesis.

For the actual mate choice, the difference between our result and Todd et al.’s [24] result may arise from two possible reasons. Firstly, in Todd et al.’s [24] study, men and women have only five minutes to talk to each other before they made the decision. Obviously, for the long-term mate choice, the five minutes should be not enough. Therefore, the participants may use the potentials-attract rule as a short-term mating strategy in a speed-dating event. Secondly, in Todd et al.’s [24] study, men’s and women’s mate choice were independent of each other, i.e. when a man (or woman) chose a woman (or man), he (or she) did not need to consider whether this women (or man) also likes him (or her). This may also result in that the participants use the potentials-attract rule in Todd et al.’s [24] study. In addition, the cultural difference may be also an important reason since some authors have shown people in a collectivist society tend to maintain longer relationships than individualists do [30].

As pointed out by Buston and Emlen [19], if people indeed are making mate choices following the likes-attract rule as an evolved mate-choice mechanism, then we should expect the assortative mating based on a trait-by-trait way to be the most frequent form; furthermore, it will be resulting in higher reproductive success. There is some evidence supporting these two points. Some studies have shown that the majority of mates share many attributes [4], [14], [15], [16], [17], [18], [31], [32], [33], and that the mate similarity enhances marriage quality and marital stability [31], [32], [34], [35], [36], [37] (which in turn may contribute to reproductive success [20], [21]), as well as fecundity [20], [21], [38], [39], [40], [41] and offspring well-being [42].

In our analysis, only the data of the couples who reported their successful stories online were included (since we cannot access the data of the couples who had not reported their stories). This means that our results may miss unhappy couples whose choices might be or not be in line with the likes-attract rule. Hence, it might be important to compare the successful couples with the unsuccessful couples in the future study.

Although the likes-attract mechanism mainly determined the human mate choice in modern China, we also found that the potentials-attract hypothesis still partially worked. For example, women’s stated income preference were partially influenced by their own height and self-rated physical attractiveness, i.e. if a woman is tall or she thinks that her own physical attractiveness is good, she may more prefer a man with high income level; and men’s age preference could be partially explained by their own income, education and desire for children, i.e. a man with higher income level, good education background and strong desire for children may prefer younger women. For the relationship between individuals’ own attributes and their actual mate choice, we also noticed that the effect of women’s self-perception of their own physical attractiveness on their partners’ income is almost equal to the effect of their own self-rated physical attractiveness on their partners’ self-rated physical attractiveness. These results seem to match the potentials-attract hypothesis. In addition, we found that women’s income also positively correlated with men’s self-rated physical attractiveness, i.e. women also use their income to get more attractive men etc. This phenomenon was also found by Butson and Emlen [19] in their study. Similarly, the correlation between women’s education level and their age preference was negative. This may imply that women with better education background would like also to find a younger mate, just like men do.

In the section of data and methods, we have pointed out that in our study both men’s and women’s physical attractiveness was measured by their self-ratings. Individuals’ self-rated physical attractiveness stands for their self-perception of their own physical attractiveness, and it may influence their mate preference and mate choice. However, the self-rated physical attractiveness has been considered as a self-concept (even self-esteem) rather than a valid measure of actual physical attractiveness [27]. Recently, Weeden and Sabini [43] examined whether the self-ratings of attractiveness are significantly related to the third-party ratings. They found that the standard objective measures could predict about 25% variations in the self-ratings of physical attractiveness [43], i.e. the self-ratings of physical attractiveness should be positively related to the objective measure of physical attractiveness. Therefore, for the physical attractiveness, although our results only show the effects of individuals’ self-perception on their mate preference and their actual mate choice, it still partially reflected the effect of the objective physical attractiveness.

In this study, two previous studies [19], [24] were compared on the issue of how individuals’ mate preference and actual mate choice are influenced by their self-perceptions. For human long-term mate choice in modern China, our main results are more consistent with the likes-attract hypothesis than the potentials-attract hypothesis. However, we also noticed that the potentials-attract hypothesis still partially works, i.e. a few variations in mate preference and actual mate choice could be explained by the potentials-attract hypothesis. Our research highlights the importance of studying human actual mate choice under different cultural backgrounds.

## Supporting Information

Figure S1 **Location distribution of the Baihe website’s users.** X-axis indicates 31 provinces in mainland China sorted by Gross Domestic Product (GDP) in 2010 in descending order. Three panels indicate that (a) all users of the website; (b) paired couples via the website; and (c) active users.
(TIF)

Figure S2 **Age distribution of the Baihe website’s users.** Three panels indicate that (a) all users of the website; (b) paired couples via the website; and (c) active users.
(TIF)

Table S1 **The profile characteristics that users could specify about themselves.**
(DOC)

Table S2 **The profile characteristics that users could specify about the partners they would like to meet.**
(DOCX)

Table S3 **Sample demographics of the data of paired couples (women: n = 590; men: n = 590).**
(DOC)

Table S4 **Sample demographics of the data of active users (women: n = 13,087, men: n = 12,916).**
(DOC)

Table S5 **Results from univariate linear regressions of stated preferences on personal information for women.**
(DOCX)

Table S6 **Results from univariate linear regressions of stated preferences on personal information for men.**
(DOCX)

Table S7 **Results from univariate linear regressions of partners’ attributes on individuals’ own attributes.**
(DOCX)
