# Supplementary figures and images for: Potentials-Attract or Likes-Attract in Human Mate Choice in China
Source: PLoS One. 2013 Apr 2;8(4):e59457. doi: 10.1371/journal.pone.0059457 (PMC3615121; doi:10.1371/journal.pone.0059457)

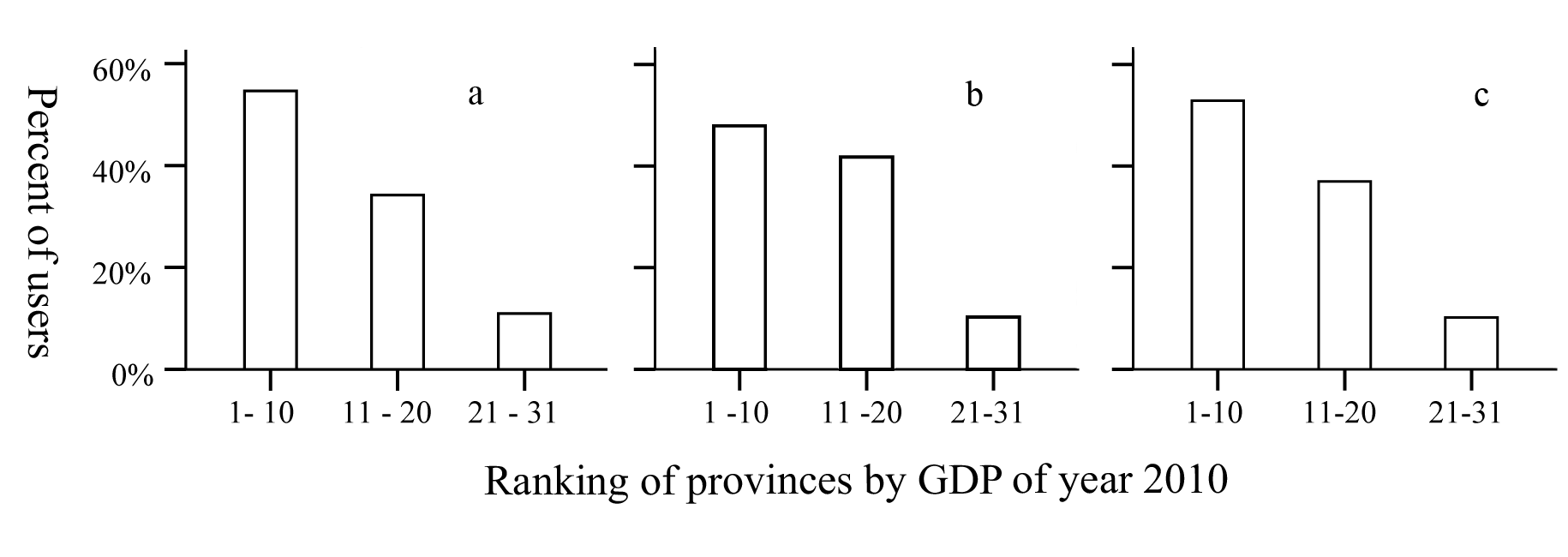

Supplement: Figure S1 — Location distribution of the Baihe website’s users. X-axis indicates 31 provinces in mainland China sorted by Gross Domestic Product (GDP) in 2010 in descending order. Three panels indicate that (a) all users of the website; (b) paired couples via the website; and (c) active users. (TIF) [file pone.0059457.s001.tif]

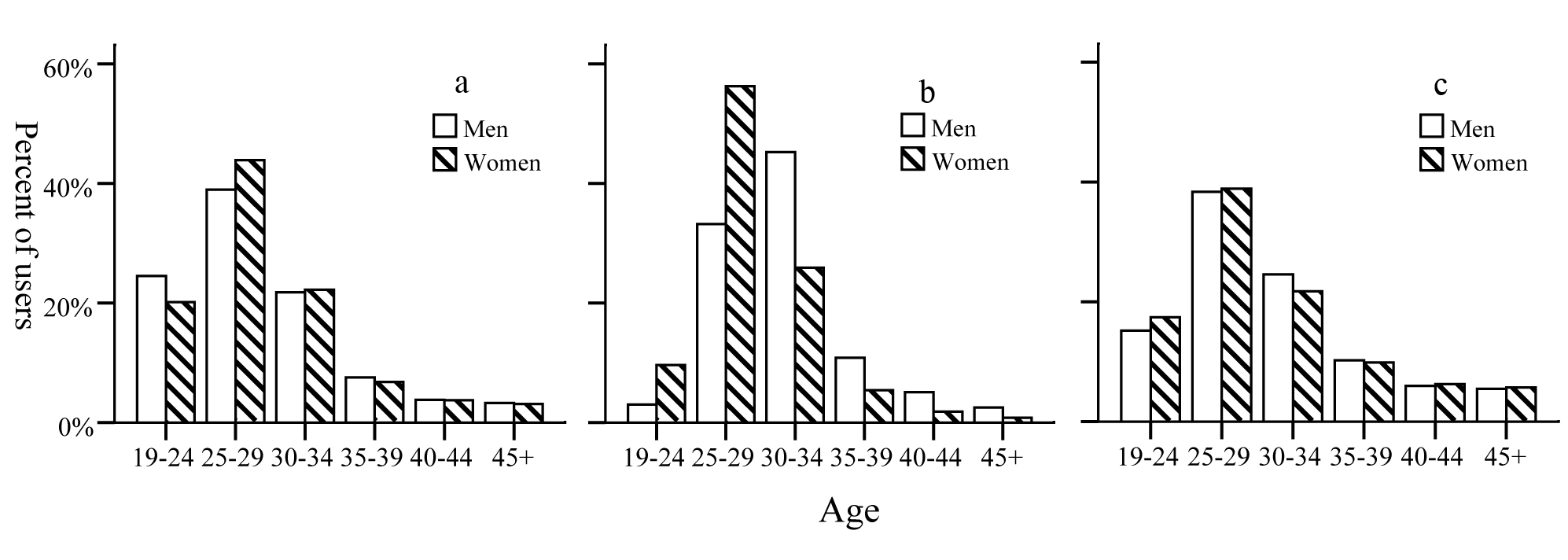

Supplement: Figure S2 — Age distribution of the Baihe website’s users. Three panels indicate that (a) all users of the website; (b) paired couples via the website; and (c) active users. (TIF) [file pone.0059457.s002.tif]
