# Supplementary material for: Potentials-Attract or Likes-Attract in Human Mate Choice in China
Source: PLoS One. 2013 Apr 2;8(4):e59457. doi: 10.1371/journal.pone.0059457 (PMC3615121; doi:10.1371/journal.pone.0059457)
Supplement: Table S1 — The profile characteristics that users could specify about themselves. (DOC) [file pone.0059457.s003.doc]

**Table S1. The profile characteristics that users could specify about themselves.**

| Traits | Description | Type |
| --- | --- | --- |
| Location | City, Province | Required |
| Gender | Male, Female | Required |
| Birth year | 1925-1992 | Required |
| Height | Less than 145 cm, 145- 195 cm, More than 195 cm | Required |
| Weight | Less than 40 kg, 40-120 kg , More than 120 kg | Optional |
| Photo | Yes/No ( there could be more than one photo for a user) | Optional |
| Income level (monthly) | Less than2000 RMB, 2000-5000 RMB, 5000-10000 RMB, 10000-15000 RMB, 15000-20000 RMB, 20000-30000 RMB, More than 30000 RMB | Required |
| Education level | High school or below, Bachelor, Master, Doctor | Required |
| House condition | Renting a flat, Living with parents, Living in company, Living in kin’s or friend’s flat, Homeowner | Optional |
| Children | Living with me, Sometimes living with me, Living apart from home, Don’t have any | Required |
| Marital status | Single, Divorced, Widowered, Married | Required |
| Self-rated physical attractiveness | 1-10 | Optional |
| Desires for children | Yes, No, Not sure | Optional |
| Occupation |  | Optional |
| Hometown | City, Province | Optional |
| Love type | 16 types from MBTI | Optional |
| Ethnicity | Han, Mongolian, Hui, Tibetan, Uighur, Hmong, Yi, Zhuang, Buyi, Korean，Manchu, Others | Optional |
| Is the only child in family | Yes, No | Optional |
| Blood type | A, B, AB, O | Optional |
| Chinese Zodiac | Rat, ox, tiger, rabbit, dragon, snake, horse, goat, monkey, rooster, dog, pig | Optional |
| Zodiac sign | Capricorn, Aquarius, Pisces, Aries, Taurus, Gemini, Cancer, Leo, Virgo, Lira, Scorpio, Sagittarius | Optional |
| School |  | Optional |
| Major |  | Optional |
| Body type | Very thin, Thin, Slim, Symmetry, Tall, Bosomy，Strong, A little fat, Fat | Optional |
| Religion |  | Optional |
| Property |  | Optional |
| Enterprise Property |  | Optional |
| Industry |  | Optional |
