# Supplementary material for: Potentials-Attract or Likes-Attract in Human Mate Choice in China
Source: PLoS One. 2013 Apr 2;8(4):e59457. doi: 10.1371/journal.pone.0059457 (PMC3615121; doi:10.1371/journal.pone.0059457)
Supplement: Table S2 — The profile characteristics that users could specify about the partners they would like to meet. (DOCX) [file pone.0059457.s004.docx]

**Table S2. The profile characteristics that users could specify about the partners they would like to meet.**

| Preferences | Description | Type |
| --- | --- | --- |
| Age preference (minimum and maximum) | 18-65 years, More than 65 years | Required |
| Location | City, Province | Required for province; optional for city |
| Height preference (minimum and maximum) | Less than 145cm, 145-195cm, More than 195cm | Required |
| Education preference | No requirement, High school or below, University, Master, Doctor | Required but multiple selection allowed |
| Income preference | No requirement, Less than 2000 RMB, 2000-5000 RMB, 5000-10000 RMB, 10000-15000 RMB, 15000-20000 RMB, 20000-30000 RMB, More than 30000 RMB | Required but multiple selection allowed |
| Does user prefer a homeowner | No requirement, Renting a flat, Living with parents, Living in company, Living in kin’s or friend’s flat, Homeowner | Optional |
| Does user prefer a mate never married | No requirement, Single, Divorced, Widower | Required but multiple selection allowed |
| Does user prefer a mate has no children | No requirement, Living with me, Sometimes living with me, Living apart from home, Don’t have one | Required but multiple selection allowed |
