# Supplementary material for: Potentials-Attract or Likes-Attract in Human Mate Choice in China
Source: PLoS One. 2013 Apr 2;8(4):e59457. doi: 10.1371/journal.pone.0059457 (PMC3615121; doi:10.1371/journal.pone.0059457)
Supplement: Table S3 — Sample demographics of the data of paired couples (women: n = 590; men: n = 590). (DOC) [file pone.0059457.s005.doc]

**Table S3. Sample demographics of the data of paired couples (women: n = 590; men: n** = 590).

| Attributes | Women | Men |
| --- | --- | --- |
| Age (SD) | 28.90 (4.20) | 31.59 (4.82) |
| Height (SD) | 163.18 (5.59) | 175.26(5.88) |
| Self-rated physical attractiveness (SD) | 7.08 (1.86) | 7.11 (1.85) |
| Income |  |  |
| Less than 2000 RMB | 36.8% | 11.9% |
| 2000-5000 RMB | 51.5% | 50.8% |
| 5000-10000 RMB | 8.6% | 28.0% |
| 10000-15000 RMB | 1.4% | 3.6% |
| 15000-20000 RMB | 1.2% | 2.5% |
| 20000-30000 RMB | 0.3% | 1.4% |
| More than 30000 RMB | 0.2% | 1.9% |
| Education level |  |  |
| High school or below | 11.4% | 7.5% |
| Bachelor | 82.9% | 80.7% |
| Master’s degree or master candidate | 5.4% | 9.8% |
| Doctoral degree or doctor candidate | 0.3% | 2.0% |
| Desire for children |  |  |
| Don’t want children | 3.7% | 3.2% |
| Not sure | 31.0% | 27.3% |
| Want children | 65.3% | 69.5% |
| Minimum Age preference (SD) | 24.26 (4.36) | 24.46 (4.61) |
| Minimum Height preference (SD) | 165.04 (8.21) | 165.99 (8.13) |
| Minimum income preference (Median) | Less than 2000 RMB | Less than 2000 RMB |
| Minimum education preference (Median) | Bachelor | Bachelor |

Notes: The range of self-rated physical attractiveness was 1-10 point.
