# Supplementary material for: Potentials-Attract or Likes-Attract in Human Mate Choice in China
Source: PLoS One. 2013 Apr 2;8(4):e59457. doi: 10.1371/journal.pone.0059457 (PMC3615121; doi:10.1371/journal.pone.0059457)
Supplement: Table S4 — Sample demographics of the data of active users (women: n = 13,087, men: n = 12,916). (DOC) [file pone.0059457.s006.doc]

**Table S4. Sample demographics of the data of active users (women: n = 13,087, men: n** = 12,916).

| Attributes | Women | Men |
| --- | --- | --- |
| Age (SD) | 30.46(7.12) | 30.68 (6.88) |
| Height (SD) | 162.68(4.60) | 174.1(5.16) |
| Self-rated physical attractiveness (SD) | 7.06 (1.70) | 6.88 (1.78) |
| Income |  |  |
| Less than 2000 RMB | 31.9% | 16.0% |
| 2000-5000 RMB | 54.0% | 52.2% |
| 5000-10000 RMB | 11.5% | 20.7% |
| 10000-15000 RMB | 1.5% | 5.1% |
| 15000-20000 RMB | 0.6% | 2.2% |
| 20000-30000 RMB | 0.2% | 1.6% |
| More than 30000 RMB | 0.3% | 2.1% |
| Education level |  |  |
| High school or below | 15.6% | 16.8% |
| Bachelor | 78.0% | 73.4% |
| Master’s degree or master candidate | 5.8% | 8.6% |
| Doctoral degree or doctor candidate | 0.5% | 1.1% |
| Desire for children |  |  |
| Don’t want children | 9.8% | 4.2% |
| Not sure | 27.5% | 26.5% |
| Want children | 62.7% | 69.2% |
| Minimum Age preference (SD) | 29.53 (6.85) | 22.68 (4.41) |
| Minimum Height preference (SD) | 171.63 (3.93) | 158.38(4.39) |
| Minimum income preference (Median) | 2000 to 5000 RMB | Less than 2000 RMB |
| Minimum education preference (Median) | Bachelor | High school or below |

Notes: The range of Self-rated physical attractiveness was 1-10 point.
