# Supplementary material for: Potentials-Attract or Likes-Attract in Human Mate Choice in China
Source: PLoS One. 2013 Apr 2;8(4):e59457. doi: 10.1371/journal.pone.0059457 (PMC3615121; doi:10.1371/journal.pone.0059457)
Supplement: Table S5 — Results from univariate linear regressions of stated preferences on personal information for women. (DOCX) [file pone.0059457.s007.docx]

**Table S5. Results from univariate linear regressions of stated preferences on personal information for women.**

| Women | | Stated preference | | | |
| --- | --- | --- | --- | --- | --- |
|  |  | Age | Height | Income | Education |
| Age | Beta weight | 0.9054 | -0.1791 | 0.0525 | 0.0100 |
|  | Adjusted R^2^ | 0.8198 | 0.0320 | 0.0027 | 0.0000 |
|  | P | **0.0000** | **0.0000** | **0.0000** | 0.2404 |
| Height | Beta weight | -0.0531 | 0.4127 | 0.0810 | 0.0511 |
|  | Adjusted R^2^ | 0.0027 | 0.1703 | 0.0065 | 0.0025 |
|  | P | **0.0000** | **0.0000** | **0.0000** | **0.0000** |
| Self-attract^a^ | Beta weight | 0.1078 | 0.0319 | 0.1165 | 0.0777 |
|  | Adjusted R^2^ | 0.0115 | 0.0009 | 0.0135 | 0.0060 |
|  | P | **0.0000** | **0.0002** | **0.0000** | **0.0000** |
| Income | Beta weight | 0.1368 | 0.0206 | 0.2951 | 0.1160 |
|  | Adjusted R^2^ | 0.0187 | 0.0004 | 0.0870 | 0.0134 |
|  | P | **0.0000** | **0.0159** | **0.0000** | **0.0000** |
| Education | Beta weight | -0.0077 | 0.0678 | 0.0898 | 0.4025 |
|  | Adjusted R^2^ | 0.0000 | 0.0045 | 0.0080 | 0.1619 |
|  | P | 0.3683 | **0.0000** | **0.0000** | **0.0000** |
| Desire for children | Beta weight | -0.4545 | 0.0326 | 0.0219 | 0.0702 |
|  | Adjusted R^2^ | 0.2065 | 0.0010 | 0.0004 | 0.0049 |
|  | P | **0.0000** | **0.0001** | **0.0106** | **0.0000** |

N =13677. Significant *P*-values are indicated in bold.

^a^. Self-attract refers to self-rated physical attractiveness.
