# Supplementary material for: Potentials-Attract or Likes-Attract in Human Mate Choice in China
Source: PLoS One. 2013 Apr 2;8(4):e59457. doi: 10.1371/journal.pone.0059457 (PMC3615121; doi:10.1371/journal.pone.0059457)
Supplement: Table S6 — Results from univariate linear regressions of stated preferences on personal information for men. (DOCX) [file pone.0059457.s008.docx]

**Table S6. Results from univariate linear regressions of stated preferences on personal information for men.**

| Men | | Stated preference | | | |
| --- | --- | --- | --- | --- | --- |
|  |  | Age | Height | Income | Education |
| Age | Beta weight | 0.7589 | -0.0825 | 0.0669 | 0.0295 |
|  | Adjusted R^2^ | 0.5759 | 0.0067 | 0.0044 | 0.0008 |
|  | P | **0.0000** | **0.0000** | **0.0000** | **0.0006** |
| Height | Beta weight | -0.0721 | 0.3805 | 0.0548 | 0.0773 |
|  | Adjusted R^2^ | 0.0051 | 0.1447 | 0.0029 | 0.0059 |
|  | P | **0.0000** | **0.0000** | **0.0000** | **0.0000** |
| Self-attract^a^ | Beta weight | 0.0056 | 0.0393 | 0.0448 | 0.0705 |
|  | Adjusted R^2^ | 0.0000 | 0.0015 | 0.0019 | 0.0049 |
|  | P | 0.5132 | **0.0000** | **0.0000** | **0.0000** |
| Income | Beta weight | 0.0913 | 0.0635 | 0.1158 | 0.1296 |
|  | Adjusted R^2^ | 0.0083 | 0.0040 | 0.0133 | 0.0167 |
|  | P | **0.0000** | **0.0000** | **0.0000** | **0.0000** |
| Education | Beta weight | 0.0226 | 0.0737 | 0.0963 | 0.3431 |
|  | Adjusted R^2^ | 0.0004 | 0.0054 | 0.0092 | 0.1177 |
|  | P | **0.0086** | **0.0000** | **0.0000** | **0.0000** |
| Desire for children | Beta weight | -0.2465 | 0.0302 | 0.0063 | 0.0715 |
|  | Adjusted R^2^ | 0.0607 | 0.0008 | 0.0000 | 0.0050 |
|  | P | **0.0000** | **0.0004** | 0.4633 | **0.0000** |

N =13506. Significant *P*-values are indicated in bold.

^a^ Self-attract refers to self-rated physical attractiveness.
