# Supplementary material for: Potentials-Attract or Likes-Attract in Human Mate Choice in China
Source: PLoS One. 2013 Apr 2;8(4):e59457. doi: 10.1371/journal.pone.0059457 (PMC3615121; doi:10.1371/journal.pone.0059457)
Supplement: Table S7 — Results from univariate linear regressions of partners’ attributes on individuals’ own attributes. (DOCX) [file pone.0059457.s009.docx]

**Table S7. Results from univariate linear regressions of partners’ attributes on individuals’ own attributes.**

|  | | | Men's attributes | | | | | |
| --- | --- | --- | --- | --- | --- | --- | --- | --- |
|  |  |  | Age | Height | Self-attract^a^ | Income | Education | Desire for children |
| Women’s attributes | Age | Beta weight | 0.6185 | -0.1179 | 0.0599 | 0.0656 | 0.0313 | -0.1385 |
|  |  | Adjusted R^2^ | 0.3815 | 0.0122 | 0.0019 | 0.0026 | -0.0007 | 0.0175 |
|  |  | P | **0.0000** | **0.0041** | 0.1458 | 0.1116 | 0.4472 | **0.0007** |
|  | Height | Beta weight | -0.0650 | 0.1869 | -0.0787 | 0.0487 | -0.0048 | 0.0639 |
|  |  | Adjusted R^2^ | 0.0025 | 0.0333 | 0.0045 | 0.0007 | -0.0017 | 0.0024 |
|  |  | P | 0.1148 | **0.0000** | 0.0562 | 0.2372 | 0.9076 | 0.1208 |
|  | Self-attract^a^ | Beta weight | 0.1018 | -0.0477 | 0.1145 | 0.1110 | 0.0765 | -0.0195 |
|  |  | Adjusted R^2^ | 0.0087 | 0.0006 | 0.0114 | 0.0106 | 0.0042 | -0.0013 |
|  |  | P | **0.0133** | 0.2473 | **0.0054** | **0.0070** | 0.0633 | 0.6368 |
|  | Income | Beta weight | 0.1618 | 0.0411 | 0.1074 | 0.3109 | 0.1093 | 0.0457 |
|  |  | Adjusted R^2^ | 0.0245 | 0.0000 | 0.0099 | 0.0951 | 0.0103 | 0.0004 |
|  |  | P | **0.0001** | 0.3188 | **0.0090** | **0.0000** | **0.0079** | 0.2676 |
|  | Education | Beta weight | 0.0360 | 0.0539 | 0.0721 | 0.0760 | 0.2486 | -0.0408 |
|  |  | Adjusted R^2^ | -0.0004 | 0.0012 | 0.0035 | 0.0041 | 0.0602 | 0.0000 |
|  |  | P | 0.3828 | 0.1908 | 0.0802 | 0.0652 | **0.0000** | 0.3227 |
|  | Desire for children | Beta weight | -0.0861 | 0.0147 | -0.0291 | 0.0476 | 0.0463 | 0.1497 |
|  |  | Adjusted R^2^ | 0.0057 | -0.0015 | -0.0009 | 0.0006 | 0.0004 | 0.0207 |
|  |  | P | 0.0366 | 0.7210 | 0.4803 | 0.2479 | 0.2616 | **0.0003** |

N =590. Significant *P*-values are indicated in bold.

^a^. Self-attract refers to self-rated physical attractiveness.
